# Identification of new biomarkers and immune infiltration characteristics of sepsis in very low birth weight infants

**DOI:** 10.17305/bb.2023.8966

**Published:** 2023-10-01

**Authors:** Yujia Luo, Zhou Jiang, Rui Gu, Xuandong Zhang, Li Wei, Yuanyuan Zhou, Songying Zhang

**Affiliations:** 1 Department of NICU, Sir Run Run Shaw Hospital, Zhejiang University School of Medicine, Qiantang District, Hangzhou, China; 2 Department of Reproductive Endocrinology, Women’s Hospital, Zhejiang University School of Medicine, Hangzhou, China; 3 Department of Obstetrics and Gynecology, Sir Run Run Shaw Hospital, Zhejiang University School of Medicine, Shangcheng District, Hangzhou, China

**Keywords:** Sepsis, very low birth weight (VLBW) infants, weighted gene co-expression network analysis (WGCNA), immune infiltration, biomarker

## Abstract

Sepsis is a life-threatening condition, especially in very low birth weight (VLBW) infants, and its pathogenesis remains unclear. Effective biomarkers need to be found to diagnose and treat the disease at an early stage. The Gene Expression Omnibus (GEO) database was screened and analyzed for differentially expressed genes (DEGs) in VLBW infants with sepsis. DEGs were then analyzed for functional enrichment. A weighted gene co-expression network analysis (WGCNA) was performed to identify the key modules and genes. The optimal feature genes (OFGs) were created using three machine learning algorithms. The single-sample Gene Set Enrichment Analysis (ssGSEA) scored the degree of immune cell enrichment between septic and control patients, and the correlation between OFGs and immune cells was evaluated. A total of 101 DEGs were identified between the sepsis and control samples. DEGs were mainly associated with immune responses and inflammatory signaling pathways in the enrichment analysis. In the WGCNA analysis, the MEturquoise module was significantly correlated with sepsis in VLBW infants (cor ═ 0.57, *P* < 0.001). By intersecting OFGs derived from three machine learning algorithms, two biomarkers were identified: glycogenin 1 (*GYG1*) and resistin (*RETN*). The area under the curves of *GYG1* and *RETN* was greater than 0.97 in the testing set. The ssGSEA indicated immune cells infiltration in septic VLBW infants, and *GYG1* and *RETN* revealed close correlations with immune cells. New biomarkers offer promising insights into the diagnosis and treatment of sepsis in VLBW infants.

## Introduction

Neonatal sepsis is a disease in which pathogens invade the blood system during the neonatal period and produce toxins that cause systemic infections [1]. With a worldwide prevalence of approximately 2% and an overall mortality rate of 11%–19% [2], a neonate’s death from neonatal sepsis falls behind prematurity and asphyxia on the list of leading causes of neonatal death [3]. Infants with very low birth weight (VLBW) are preterm babies born below 1500 g. VLBW infants are more susceptible to bacterial infections because of immature immune system development, longer hospital stays, and exposure to invasive tests and therapeutic manipulations [4, 5]. In addition, the skin of VLBW infants has a weakened external antimicrobial defense barrier due to the lack of a good stratum corneum as well as fetal lipids, which also increases the risk of infection [6, 7]. Thus, preterm infants, especially VLBW infants, are at a significant disadvantage in terms of infection prevention compared to full-term infants [4, 8].

**Figure 1.**
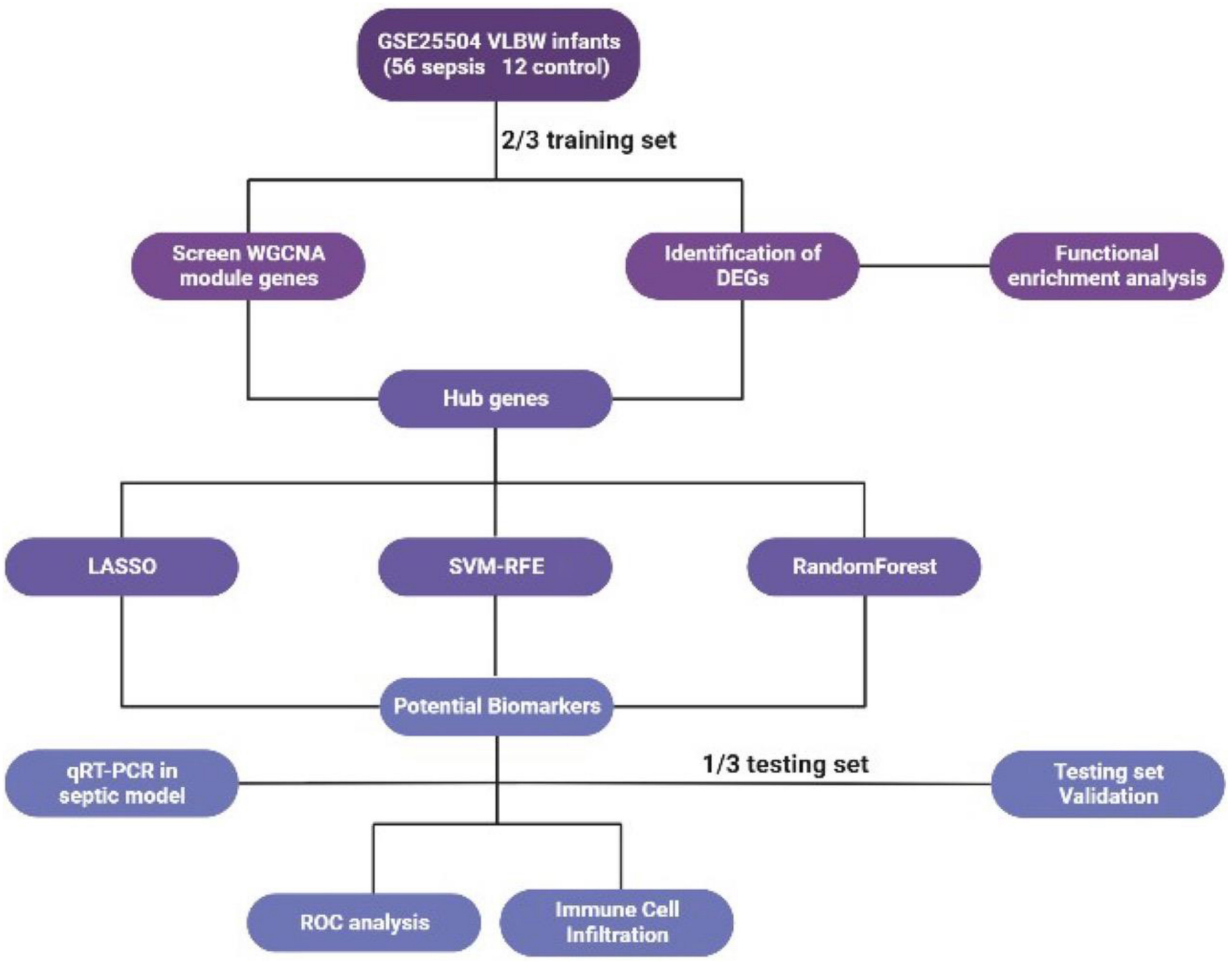
**An overview of the study’s flow diagram.** VLBW: Very low birth weight; WGCNA: Weighted gene co-expression network analysis; DEG: Differentially expressed gene; SVM-RFE: Support vector machine recursive feature elimination; qRT-PCR: Quantitative real-time PCR; ROC: Receiver operating characteristic; LASSO: Least absolute shrinkage and selection operator.

Early sepsis diagnosis in VLBW infants is still challenging for clinicians. The diagnosis of sepsis mainly relies on clinical symptoms and laboratory indicators. Due to the incompetent immune function and lack of specificity of clinical manifestations in VLBW infants, the diagnosis of sepsis cannot be clarified early. Therefore, the gold standard for detecting sepsis is blood culture. However, its low positive rate, long culture time, and impaired accuracy affected by blood volume in preterm infants, antibiotic use prior to sample collection, and maternal prenatal antibiotic use make it unsuitable for early diagnosis. In addition, commonly tested indicators of early inflammation in the neonatal intensive care unit are C-reactive protein (CRP) and calcitoninogen (PCT). Liu et al. [9] found that CRP expression levels are elevated in the early stages of inflammation and are widely used because of the ease of detection. However, CRP itself exhibits low sensitivity and specificity and is also elevated in many non-infectious conditions (e.g., perinatal asphyxia, meconium aspiration syndrome, and intraventricular hemorrhage). Simon et al. [10] found that plasma PCT changes in sepsis earlier than temperature, WBC count, and CRP and is considered an important indicator for early diagnosis and outcome evaluation. However, the diagnostic value of PCT for early-onset sepsis remains controversial due to its high fluctuation during the first three days of life [11]. Moreover, CRP and PCT are mainly produced in the liver, and there may be false-negative results for infection indicators in septic VLBW infants with immature liver development [12, 13]. In summary, neither CRP nor PCT is an ideal laboratory indicator for the early diagnosis of sepsis in VLBW infants. In addition, tumor necrosis factor and inhibitory proteins have been reported as potential biomarkers of sepsis, but the results lack reliable evidence to support them [14, 15]. Consequently, active exploration of new biomarkers is essential for the early diagnosis and prognosis of sepsis in VLBW infants.

Delano and Ward [16] reported the involvement of an activated innate immune system and a paralyzed adaptive immune system in the process of systemic inflammatory response in sepsis. Sepsis is increasingly being studied in terms of immune cell infiltration. According to Li et al.’s findings, children with sepsis had significantly fewer T cells and NK cells and significantly more neutrophils and monocytes than controls [17]. Results consistent with the above were also obtained in Huang et al.’s study that pooled adult, pediatric, and neonatal sepsis for analysis [18]. However, there are still few studies on the infiltration of immune cells in septic VLBW infants.

Accordingly, multiple bioinformatics approaches were used in our study to select key genes for sepsis in VLBW infants and validate their diagnostic performance in the testing set. Moreover, we assessed the correlation of genes with immune cells to exhibit new insights into the molecular mechanisms of the disease.

## Materials and methods

### Collection and preprocessing data

Figure 1 shows the flowchart of our study. The gene expression profiles of datasets GSE25504 were obtained from the Gene Expression Omnibus (GEO) database [19]. GSE25504 was based on GPL570, GPL13667, GPL15158, and GPL6947. In this dataset, we extracted data from 56 patients with sepsis and 12 patients without sepsis whose birth weight was less than 1500 g. The whole blood used for gene analysis was obtained at the onset of the first clinical signs of suspected sepsis. We extracted the postmenstrual age of the VLBW infants, and the mean age (weeks) at the sampling time was 31.09 (24.14∼39.86) weeks in the sepsis group and 32.75 (29.42∼37.57) weeks in the control group. By applying the packages “limma” and “sva” in R (version 4.2.2), data from different platforms were combined, and batch corrections were executed by “ComBat” package [20]. Prior to further analysis, we randomly assigned patients to the sepsis and control groups in a ratio of 2:1 by R language. Forty-five VLBW infants were assigned to the training set, and 23 were selected for the testing set.

### Gene expression and functional enrichment analyses

The package “limma” was adopted to explore gene expression differences between septic and control preterm infants. Genes with a |log2FC| >1 and a *P* value < 0.05 were regarded as differentially expressed. Heatmaps and volcano plots of differentially expressed genes (DEGs) were generated using R packages “pheatmap” and “ggplot2.” An analysis of DEG biological functions was conducted using the R package “clusterProfiler” for Gene Ontology (GO) and Kyoto Encyclopedia of Genes and Genomes (KEGG) pathway enrichment.

### Weighted gene co-expression network analysis (WGCNA)

Co-expression networks of the GSE25504 dataset were performed using the WGCNA method based on scale-free topology. Calculations of soft threshold power and adjacencies were performed using the pickSoftThreshold function of the “WGCNA” package. A topological overlap matrix was generated using the adjacency matrix, and a dissimilarity calculation was performed to determine hierarchical clustering results. A dynamic tree-cutting method was used to identify co-expressed gene modules with a minimum module size of 10. We determined the key module associated with septic neonates by measuring gene significance (GS) values and module membership (MM) values.

### Identifying optimal feature genes (OFGs)

By using the R package “glmnet,” the LASSO binary logistic regression model was used to select OFGs in the training datasets. An optimal penalty parameter was determined for each signature from a cross-validation minimum of 10 times [21]. R packages “e1071,” “kernlab,” and “caret” were used to determine the OFGs based on a support vector machine recursive feature elimination (SVM-RFE) algorithm based on a nonlinear SVM [22]. Random forest was used to generate 500 trees for each datapoint with the meanDecreaseGini score >2 considered as an OFG [23]. Protein–protein interaction (PPI) networks were predicted using the STRING (http://string-db.org).

### Diagnostic evaluation of key genes

For displaying the expression of key genes in septic and control VLBW infants, the R packages “ggplot2” and “ggpubr” were applied to create the box plots. Receiver operating characteristic (ROC) curves were calculated using the package “pROC,” and AUC was used to measure the predictive value of key genes. A gene was considered diagnostic in the training and test set if its AUC exceeded 0.85.

### Immune cell infiltration

Immune cells enriched in the samples were scored utilizing the single-sample Gene Set Enrichment Analysis (ssGSEA) method by R package “gsva.” The results were visualized using heatmaps and violin plots generated with R packages “corrplot” and “ggplot2.” Spearman correlation coefficients were used with the R statistical package to assess the associations between significant genes and immune infiltrating cells.

### Cell culture and stimulation

The experiments were conducted using human umbilical vein endothelial cells (HUVECs) (ATCC, USA) in order to mimic sepsis condition in which endothelial cells are involved in inflammation. RPMI 1640 (Gibco, USA) medium containing 10% fetal bovine serum and 1% penicillin–streptomycin was used to culture HUVECs. The cells were cultured at 37 ^∘^C, 5% CO_2_, and saturating humidity with passages 5–7. The sepsis model was created by stimulating HUVECs for 6 h with LPS (1 ug/mL, Sigma, USA), and then harvesting the cells.

### Quantitative real-time PCR (qRT-PCR)

An RNA-Quick Purification kit (Qiagen 74034, Germany) was used to extract total RNA from HUVECs, which was then reverse transcribed to cDNA using an RT-PCR Kit (A3500, Promega, USA). For the real-time PCR, cDNA was combined with SYBR Green Master Mix (DBI-2044, Germany). All primers used for the quantified PCR are listed in Table 1. *GAPDH* acted as an internal control. Expression levels of OFGs were calculated by applying 2^−ΔΔCt^ method.

**Table 1:** Primers used in quantitative real-time PCR

**Primers**	**Sequence (5′-> 3′)**
*GYG1*	Forward	5′-TGACACTAACCACAAACGATGC-3′
	Reverse	5′-TAGATGAGCAGAATCGCCACT-3′
*RETN*	Forward	5′-CTGTTGGTGTCTAGCAAGACC-3′
	Reverse	5′-CCAATGCTGCTTATTGCCCTAAA-3′
*GAPDH*	Forward	5′-GCCTCAAAATCCTCTCGTTGTG-3′
	Reverse	5′-GGAAGATGGTGATGGGATTTC-3′

GYG1: Glycogenin 1; RETN: Resistin.

### Ethical statement

GEO belongs to public databases. The patients involved in the database have obtained ethical approval. Users can download relevant data for free for research and publish relevant articles. Our study is based on open-source data, so there are no ethical issues and other conflicts of interest.

### Statistical analysis

All statistical analyses and graphics were performed with R software (version 4.2.2) and *GraphPad* Prism 7 (GraphPad). *P* values < 0.05 were used to determine statistical significance.

## Results

### Determination of differentially expressed genes (DEGs)

**Figure 2.**
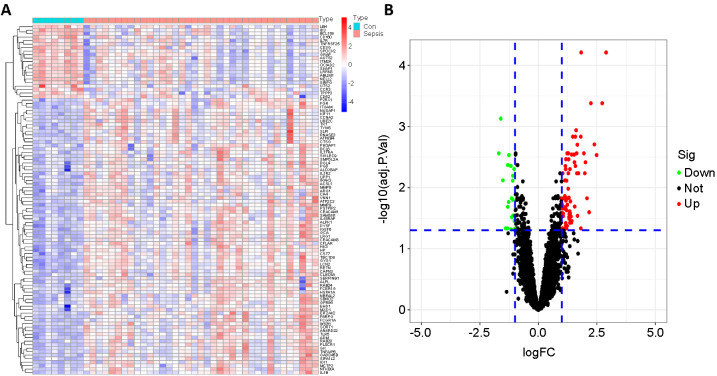
**Genes differentially expressed between septic and control (Con) infants.** There were 80 upregulated genes and 21 downregulated genes: (A) Heatmap; (B) Volcano plot.

The flow diagram of the study process is illustrated in Figure 1. DEG analysis was performed on 37 patients with sepsis and 8 controls among the training samples. Compared with the control group, the sepsis group identified 101 DEGs with 80 upregulated genes and 21 downregulated genes. Significant results are displayed using heatmaps and volcano plots (Figure 2).

### Enrichment of differentially expressed genes (DEGs) for function

**Figure 3.**
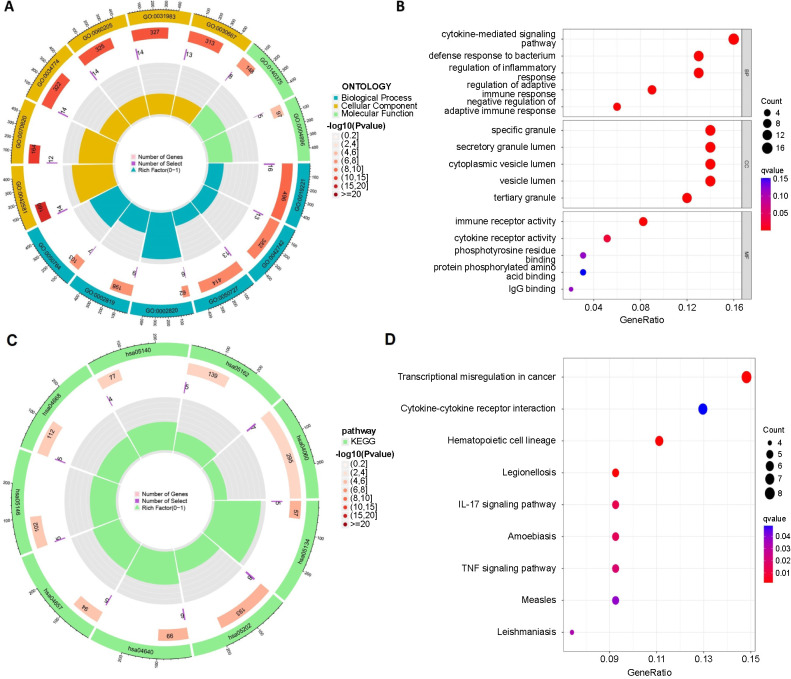
**Enrichment of DEGs for function.** (A and B) Forecasts of DEG functions were achieved using GO analysis. The cytokine-mediated signaling pathway, specific granule, and immune receptor activity were functionally enriched in septic neonates; (C and D) KEGG pathway was evaluated regarding DEGs. The immune-related pathways were enriched in septic neonates. DEG: Differentially expressed gene; GO: Gene Ontology; KEGG: Kyoto Encyclopedia of Genes and Genomes; IL: Interleukin; TNF: Tumor necrosis factor.

DEGs were enriched for GO and KEGG pathways using the R package “clusterProfiler.” Immune and inflammatory responses accounted for the majority of the results. The top five terms in biological process (BP), cellular component (CC), and molecular function (MF) were identified in which cytokine-mediated signaling pathway, specific granule, and immune receptor activity were functionally enriched in septic neonates (Figure 3A and 3B). Among the results of KEGG analysis, there were numerous immune-related pathways, such as the IL-17 signaling pathway, cytokine–cytokine receptor interactions, and TNF signaling pathway (Figure 3C and 3D).

### Weighted gene co-expression network analysis (WGCNA)

**Figure 4.**
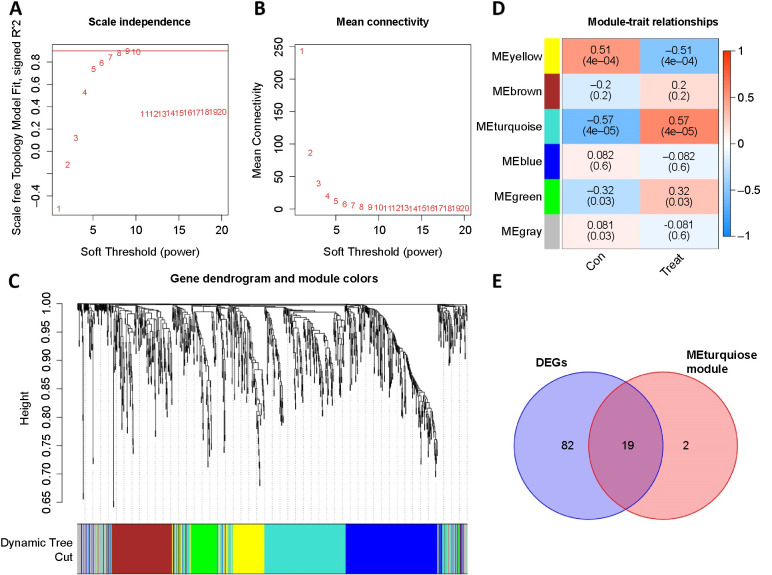
**WGCNA and identification of possible significant genes.** (A and B) WGCNA’s soft threshold power was 8 and high mean connectivity was maintained; (C and D) Clustering gene dendrogram and modules of WGCNA. The MEturquoise module showed a remarkable correlation with septic neonates (cor ═ 0.57, *P* < 0.001); (E) The interaction genes between DEGs and MEturquoise module. WGCNA: Weighted gene co-expression network analysis; DEG: Differentially expressed gene.

The expression data of the training set was calculated using package “WGCNA” in R, and a co-expression network with scale-free was constructed. With a scale-free index of 0.86 and an 8 soft threshold power, high mean connectivity was maintained (Figure 4A and 4B). Figure 4C shows the cluster dendrogram. Lastly, six modules were derived from the data (Figure 4D). The correlation between various modules and sepsis in VLBW infants was analyzed. The findings indicated that the MEturquoise module showed a remarkable correlation with sepsis in VLBW infants (cor ═ 0.57, *P* < 0.001). Figure 4E illustrates the overlap between DEGs and hub genes in MEturquoise.

### Screening optimal feature genes (OFGs)

**Figure 5.**
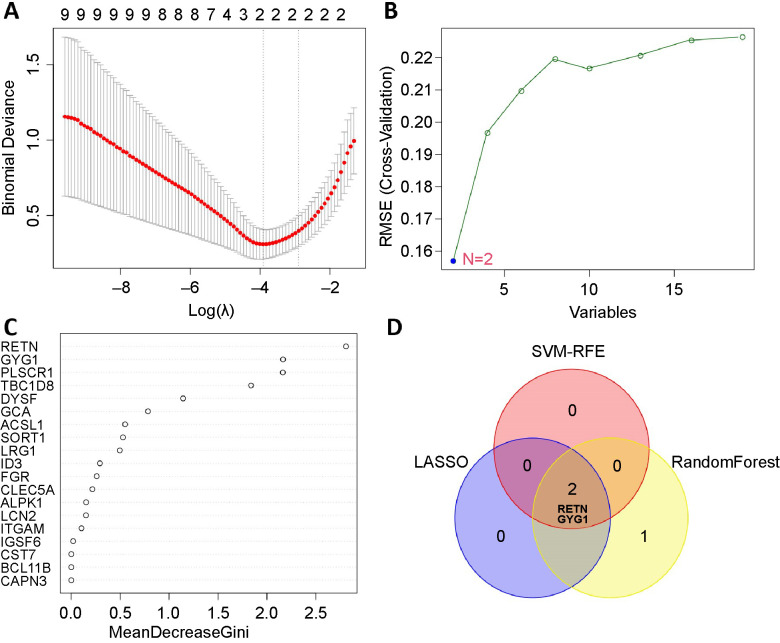
**Detection of potential OFGs in premature infant sepsis.** (A) LASSO algorithm identified two OFGs; (B) SVM-RFE algorithm selected two OFGs; (C) RandomForest algorithm determined three OFGs; (D) The OFGs interacted by three machine learning algorithms were *GYG1* and *RETN*. OFG: Optimal feature gene; SVM-RFE: Support vector machine recursive feature elimination; GYG1: Glycogenin 1; RETN: Resistin; LASSO: Least absolute shrinkage and selection operator.

For the above-overlapped genes, the LASSO algorithm was used to select two key genes, and SVM-RFE was performed to filter two functional genes. Moreover, the RF algorithm identified three significant genes (Figure 5A–5C). The two upregulated OFGs, named glycogenin 1 (*GYG1*) and resistin (*RETN*), were obtained by intersecting these genes (Figure 5D). To substantiate their role as novel biomarkers in the early detection of sepsis, the PPI network analysis of the OFGs was performed and displayed in Figure S1.

### Diagnostic evaluation of key genes.

**Figure 6.**
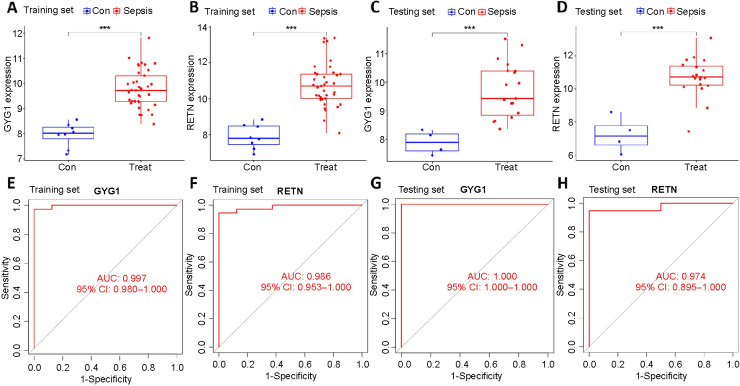
**Validation of the potential biomarkers.** (A–D) The expression of *GYG1* and *RETN* between sepsis (Treat) and control (Con) groups in the training and testing set. ***P < 0.001; (E–H) The *GYG1* and *RETN* displayed diagnostic value with AUCs > 0.95 in the training and testing set. GYG1: Glycogenin 1; RETN: Resistin; AUC: Area under the curve.

A significant increase in the expression of *GYG1* and *RETN* was observed in VLBW infants with sepsis in both the training and testing datasets (Figure 6A–6D). In addition, we established the ROC curves for genes in both datasets to check the diagnostic value. The results revealed that *GYG1* and *RETN* showed excellent diagnostic efficiency with AUCs > 0.95 in both datasets (Figure 6E–6H). Accordingly, *GYG1* and *RETN* were identified as candidate biomarkers for the diagnosis of sepsis in VLBW infants.

### Evaluation of immune cell infiltration

**Figure 7.**
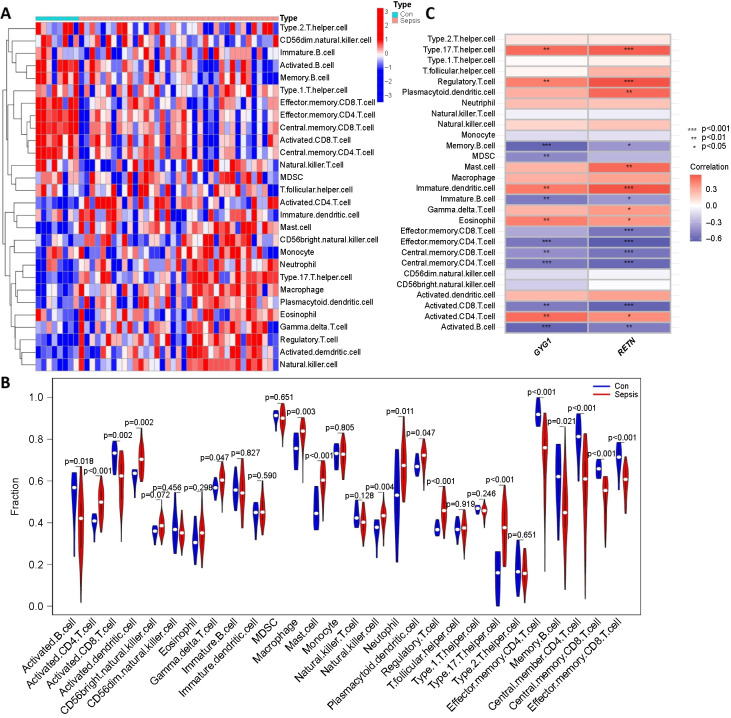
**Evaluation of immune cell infiltration.** (A) By ssGSEA score, the heatmap shows enriched immune cells in every sample; (B) A violin graph illustrates the distinct fractions of immune cells in the control (Con) and sepsis group; (C) Correlation between determined genes and immune cells. ssGSEA: single-sample Gene Set Enrichment Analysis; VLBW: Very low birth weight; GYG1: Glycogenin 1; RETN: Resistin.

Infiltration of immune cells was used to assess immunological characteristics. The heatmap showed the enrichment of immune cells in samples by ssGSEA score (Figure 7A). The violin diagram indicated that the infiltration of activated dendritic cells, activated CD4 T cells, macrophages, mast cells, neutrophils, NK cells, T helper 17 cells, and regulatory T cells was higher in sepsis patients than in controls (Figure 7B). *GYG1* and *RETN* were positively correlated with the infiltration of T helper 17 cells, regulatory T cells, immature dendritic cells, activated CD4 T cells, and eosinophils, while were negatively related to memory B cells, effector memory CD4/CD8 T cells, central memory CD4/CD8 T cells, activated CD8 T cells, and activated B cells. In addition, *RETN* was also positively correlated with gamma delta T cells and mast cells (Figure 7C).

### Validation in septic cell model

**Figure 8.**
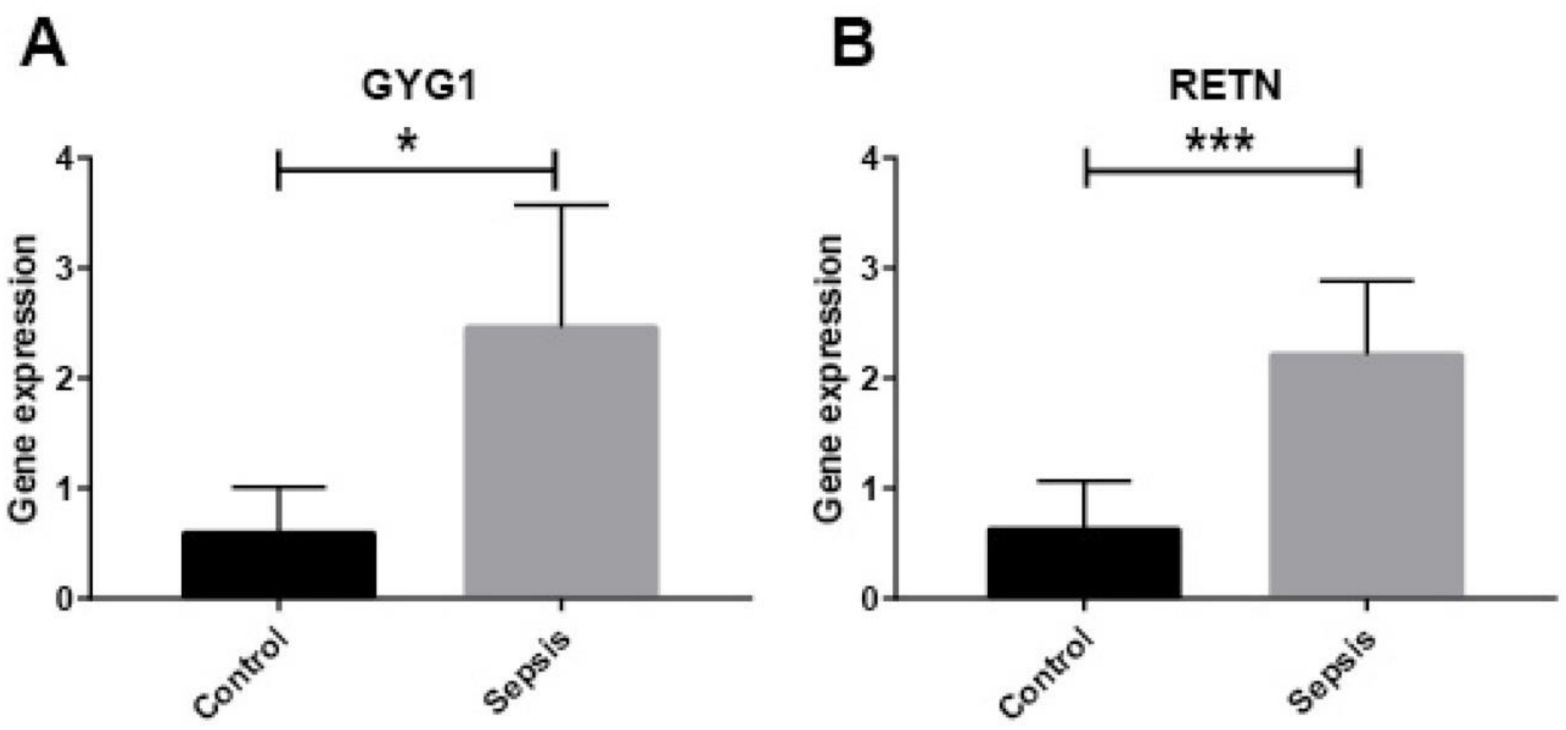
**The expression levels of two potential biomarkers were higher in septic cell model.** (A) *GYG1*; (B) *RETN*. *P < 0.05, **P < 0.01, ***P < 0.001. GYG1: Glycogenin 1; RETN: Resistin.

Using qRT-PCR, we determined the expression levels of two biomarkers in LPS-treated HUVECs in order to validate the bioinformatics results. In accordance with our bioinformatics analysis, we found that *GYG1* and *RETN* were significantly upregulated in the LPS-treated group (*P* < 0.05; Figure 8).

## Discussion

Sepsis is believed to be a life-threatening condition caused by a dysregulated inflammatory response to infection, with insidious onset and high mortality in VLBW infants [5]. Early recognition and therapy are crucial to improve the survival of VLBW infants with sepsis. Immune cell infiltration is a crucial component in the pathogenesis of infant sepsis [24]. This study assessed DEGs in VLBW infants with sepsis and controls and identified key modules based on WGCNA. The optimal signature genes for sepsis in VLBW infants in the key modules were screened by LASSO analysis, SVM-RFE algorithm, and random forest analysis, including *GYG1* and *RETN*. These signature genes’ expression levels and diagnostic values were subsequently validated in the testing set and septic cell model. Finally, the ssGSEA-based algorithm was applied to analyze the differences in immune cell infiltration between sepsis and controls in VLBW infants, and immune cells were explored for their correlation with crucial genes.

Interestingly, we identified *GYG1* and *RETN* as diagnostic markers for sepsis in VLBW infants through WGCNA analysis and machine learning algorithms. Among them, resistin (encoded by *RETN*) is a cysteine-rich small molecular protein secreted by adipocytes, which is also expressed in macrophages and neutrophils. Khattab et al. [25] found that resistin levels were significantly elevated in newborns with sepsis, septic shock, or those receiving mechanical ventilation. Moreover, resistin can also act as a proinflammatory factor, which promotes the production of proinflammatory cytokines in alveolar macrophages by mediating the TLR4/NF-κB signaling pathway [26]. Similarly, resistin inhibits neutrophil migration, bacterial clearance, and the production of reactive oxygen species, promoting the development of inflammation [27]. Therefore, *RETN* can be used as an indicator for diagnosing sepsis in VLBW infants, which is consistent with our results. Glycogenin 1 (encoded by *GYG1*) is a glucosyltransferase, a member of the glycogen protein family, expressed in skeletal muscle and liver cells and is primarily involved in the initiation of glycogen synthesis. According to recent studies, the absence of *GYG1* can cause glycogen synthesis disorders, leading to glycogen storage diseases and polysaccharide myopathy [28, 29]. However, the role of *GYG1* in sepsis in VLBW infants is unclear.

Sepsis-induced dysregulation of innate immunity and limitation of adaptive immunity together trigger persistent pro- and anti-inflammatory pathways that ultimately lead to tissue and organ dysfunction [16]. The results of our analysis showed that the proportion of innate immune cells in the sepsis group was higher, while the proportions of T cells and B cells were lower, indicating that these cells may be associated with the progression of sepsis in VLBW infants. Neutrophils are involved in the early acute inflammatory response mainly through release, migration, and phagocytosis [30]. Although neutrophils are insufficient in VLBW infants, they are still the most important line of defense against sepsis. The low activity of NK cells in newborns increases the susceptibility to sepsis. After sepsis, NK cell activity and toxicity are further decreased and are associated with disease progression and poor outcomes [31]. The results of our analysis showed that the proportion of NK cells was higher in VLBW infants with sepsis than in controls, which is inconsistent with existing conclusions and needs further confirmation. Regulatory T cells are markedly increased in patients with sepsis and correlate positively with mortality. Circulating regulatory T cells induce immunosuppression through upregulation of tumor necrosis factor receptor type 2 expression in patients with septic shock [32]. In addition, due to the insufficient acquired immunity of newborns, PD-1 exerts an immunosuppressive effect on neonatal sepsis by downregulating CD8+ T cells activity during the inflammatory response, aggravating disease progression [33]. Finally, we investigated the correlation between infiltrating immune cells and diagnostic markers. The findings revealed significant correlations between the two key genes and T helper 17 cells, regulatory T cells, immune dendritic cells, activated CD4 T cells, and eosinophils. However, little information is available on the complex interaction processes between genes and immune cells, and there is an urgent need to investigate in depth the potential molecular mechanisms and functional significance of immune cell infiltration in sepsis in VLBW infants based on the above findings.

The results of this study can help us better understand the immune-related pathogenesis of sepsis in VLBW infants and lay a molecular foundation for rapid diagnosis, drug development, and immunotherapy. However, this study also has limitations. We still need more clinical samples to verify and evaluate the reliability of the results and further explore the relevant molecular mechanisms by constructing animal models and cell experiments.

## Conclusion

This study determined the genes of key modules of sepsis in VLBW infants through WGCNA analysis and applied machine learning algorithms to screen two potential biomarkers: *GYG1* and *RETN*. In addition, this study also explored the infiltration of immune cells and its correlation with essential genes in VLBW infants with sepsis. This study not only improves the ability of early diagnosis of sepsis in VLBW infants but also provides new insights into the immunotherapy of sepsis patients.

## Supplemental Data

**Figure S1.**
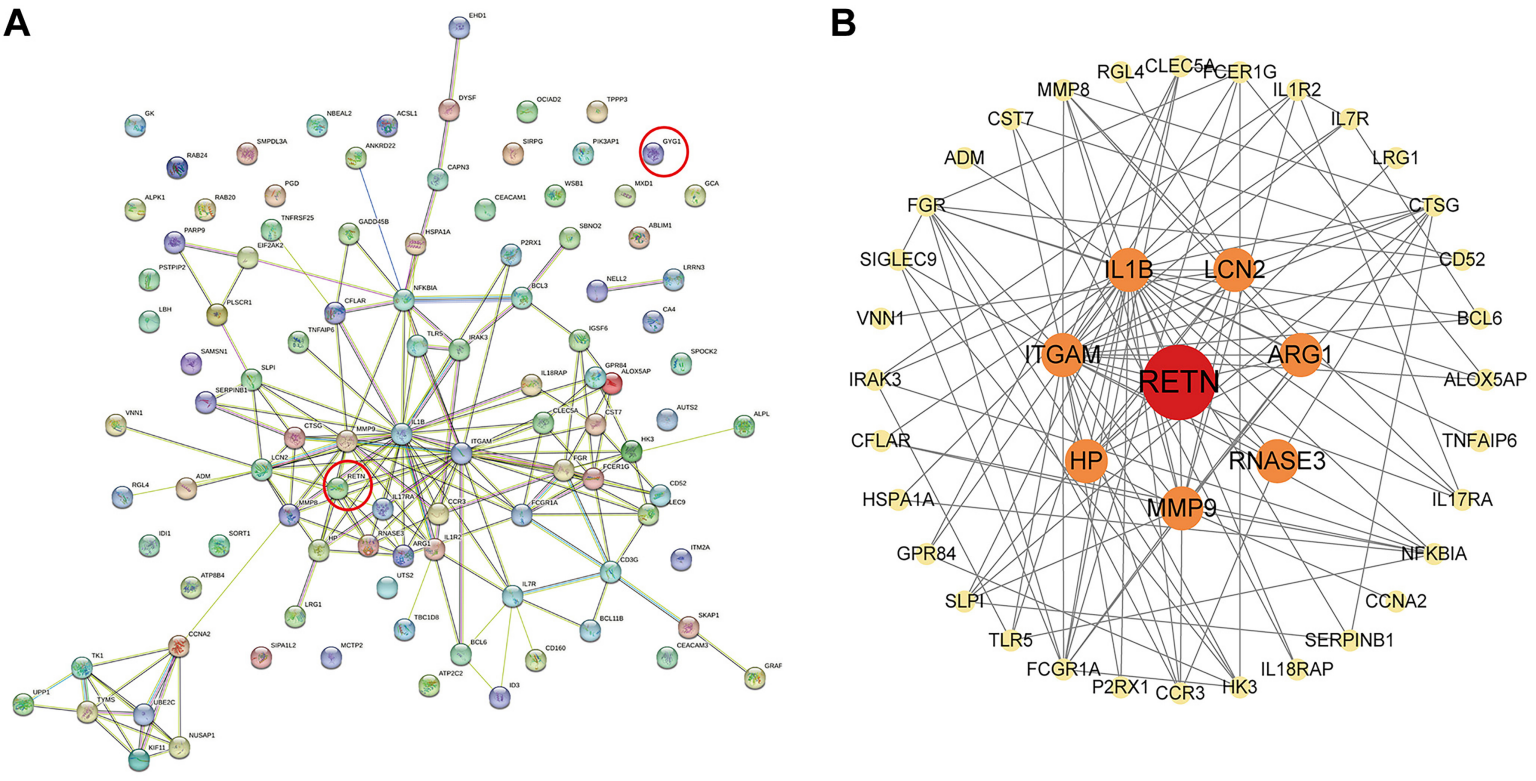
**The PPI network of the OFGs.** (A) The PPI network of DEGs. *GYG1* and *RETN* are marked by red circles. (B) The first neighbors interacting with *RETN* are marked orange and the second neighbors are marked yellow. OFG: Optimal feature gene; DEG: Differentially expressed gene; PPI: Protein-protein interaction; GYG1: Glycogenin 1; RETN: Resistin.
